# A Qualitative Study Exploring the Regional Feasibility of Patient-Reported Outcome Measures (PROMs) Data Collection for Orthopedic Trauma Patients

**DOI:** 10.7759/cureus.48906

**Published:** 2023-11-16

**Authors:** Mary E Moran, Tyler J Canova, Caleb J Hicks, Nathan R Blecker

**Affiliations:** 1 Surgery, Summa Health, Akron, USA; 2 Medicine, The University of Toledo, Toledo, USA; 3 Psychology, Kent State University, Kent, USA

**Keywords:** regional trauma system, trauma center, qualitative studies, patient reported outcomes measurement, behavioral health

## Abstract

Introduction: Behavioral health has been shown to impact both short- and long-term health outcomes in trauma patients. Recommendations for screening for behavioral health concerns in the acute setting exist, but longitudinal data collection is infrequently performed. The Trauma Quality Improvement Program describes the importance of patient-reported outcome measures (PROMs), including behavioral health data.

Methods: In this qualitative feasibility study, a multidisciplinary team participated in one-hour virtual focus groups; a semi-structured interview guide was used to ascertain feedback on a proposed PROMs study design. This study utilized a qualitative methodology to reveal thematic results from the staff feedback to determine the feasibility of the proposed study design.

Results: Three virtual one-hour focus groups consisting of a combination of seven trauma program managers and orthopedic practice managers were asked questions related to the feasibility of a PROMs study design before thematic saturation was reached. Through the analysis, four themes emerged: barriers, possible improvements, representation and research design. Themes included subthemes as well. Noteworthy results included the impact of an integrated orthopedic practice and the technological options available for use.

Conclusion: This study revealed the barriers that would exist in the implementation of PROMs for orthopedic trauma patients, which may be useful when designing data collection procedures for PROMs. The results related to barriers may assist other trauma centers or regional trauma systems in designing an optimal methodology for PROMs data. Furthermore, the American College of Surgeons might consider these results prior to any mandated implementation of PROMs for trauma centers to avoid any possible burden on staff and systems.

## Introduction

American College of Surgeons (ACS) patient-reported outcome measures

The Trauma Quality Improvement Program (TQIP) describes the importance of patient-reported outcome measures (PROMs). One of the initiatives in 2023 is a pilot study to collect PROMs data from patients [1]. The goal of PROMs is to expand the data collected on trauma patients from the acute care setting (i.e., inpatient) to including longitudinal data (i.e., post-discharge), which is not currently the standard of data collection mandated by the National Trauma Data Standard. While there is value in this additional data, the equity regarding resources and infrastructure across the various levels of trauma centers poses potential barriers to optimize such data collection.

Survey response rates

Evidence exists to demonstrate the challenges associated with patient survey response rates, particularly related to the wide range of response rates [2-5]. Researchers have reported response rates ranging from 33% to 86%, depending on the format of the survey and the response time frame [2-4]. Researchers found an average response rate of 42% for patient-reported outcome surveys related to rehabilitation with a 50% response rate for surveys administered at two weeks post-knee surgery and 33% at four weeks post-knee surgery [3]. In a similar study, patient-reported safety experience surveys were administered amongst cardiology, orthopedic, and stroke patients across four hospitals that resulted in a 16.4% response rate [4]. A systematic review of PROMs data collected for registries resulted in baseline response rates of 75% with gradual attrition during follow-up survey time points [2].

Evidence suggests that survey format can impact response rates [2,5]. Researchers found that paper format resulted in the best response rates (86%), followed by mixed electronic and paper format (71%) and only 42% resulting from electronic formats [2]. Furthermore, reminders resulted in higher response rates: one reminder had the best results (82%), followed by more than one reminder (76%) compared to no reminders with the lowest rates (39%) [2]. Interestingly, other researchers found that there was no difference between response rates from patients who chose an electronic versus a paper form of a survey in surgical patients [5].

Behavioral health in trauma

Despite the challenges associated with soliciting patient information via surveys, their role in studying behavioral health following a traumatic injury remains imperative. There is substantial literature that supports the impact of behavioral health conditions on injured patients. In fact, researchers found that up to 90% of emergency department (ED) high-utilizers had a behavioral health condition (substance use and/or mental health); however, only 10% and 17% of those patients were receiving treatment, respectively [6]. Additional literature has revealed that approximately 25% of survivors of traumatic injuries treated at Level I trauma centers received treatment for unrelated injuries within the past five years [7]. Researchers studied screening data from 2019 to 2021 for Level I and II trauma centers and found 38% of sites reported screening for depression and 28% for post-traumatic stress disorder (PTSD) [8].

Behavioral health screening in trauma

Identifying behavioral health concerns by screening reduces the risk of undetected behavioral health concerns, which has been found to impact treatment [9,10]. Researchers found associations between behavioral health conditions (i.e., depressive and PTSD symptoms) and alcohol abuse symptoms [9]. Others found relationships with behavioral health and higher self-reports of pain and lower self-reports of quality of life [9-11]. These short- and long-term implications have only recently started to be explored, as evidenced by the dearth of literature on the impact of behavioral health on trauma patients.

Staff perspectives on additional behavioral health screening

Despite the importance of behavioral health screening following injury, there is often a resistance towards the implementation of additional screening protocols from the healthcare community. Qualitative researchers revealed staff-identified barriers to consider when discussing additional psychosocial screening requirements: limitations in time, increased workload, and limitations of resources [12-14]. In fact, physician and administrative engagement were the strongest predictors of collection rates, followed by financial incentives [15]. Lack of knowledge and training in mental health were also expressed by providers as concerns, as staff are faced with time constraints and increased responsibility without any additional financial incentive [14].

The aim of this study was to explore the feasibility of PROMs data collection within a midwestern regional trauma system.

## Materials and methods

Qualitative approach and research paradigm

As this study was designed to gather expert feedback based on experience, it was identified as a phenomenological method in a qualitative study. Investigators followed the seven steps of Moustakas’ phenomenological method: (1) discovering a topic, (2) conducting a comprehensive literature review, (3) identifying appropriate co-researchers, (4) instructing co-researchers, (5) developing the interview guide, (6) conducting and recording the interviews, and (7) organizing and analyzing the data [16]. Furthermore, this feasibility study was guided by Orsmond and Cohn’s framework (Table 1) [17].

**Table 1:** Feasibility objectives and main questions

Objective	Main Question
1. Evaluation of recruitment capability and resulting sample characteristics	Can we recruit appropriate participants?
2. Evaluation and refinement of data collection procedures and outcome measures	How appropriate are the data collection procedures and outcome measures for the intended population and purpose of the study?
3. Evaluation of acceptability and sustainability of intervention and study procedures	Are study procedures and intervention suitable for and acceptable to participants?
4. Evaluation of resources and ability to manage and implement the study and intervention	Does the research team have the resources and ability to manage the study and intervention?

Research characteristics and reflexivity

The lead researcher and focus group facilitator worked at one of the regional trauma system’s Level I trauma centers as the Research Program Director. Therefore, there were previously established relationships with some of the focus group participants. This fact was acknowledged and attempts were made to reduce the risk of bias using a team approach during the data analysis phase of the study (i.e., unanimous agreement regarding coding and triangulation).

This feasibility study utilized a virtual focus group design to identify strengths and barriers of a design to implement PROMs data collection in a midwestern regional trauma system that included primarily urban trauma centers (Levels I-III).

Participants

Participant recruitment was based on trauma center membership within the regional trauma system. The invited participants included the following: trauma program managers (TPMs), orthopedic practice managers (OPMs), orthopedic providers (e.g., advanced practice providers and surgeons), and orthopedic clinic staff. This study focused on orthopedic trauma patients; therefore, there was an emphasis on feedback from the orthopedic clinic staff. Patients who suffered a fracture were more likely to have more consistent follow-up appointments (e.g., six weeks and three months post-discharge) to conduct in-person data collection via patient surveys. The feedback was collected based on the proposed PROMs study design using a multi-institution focus group with participants recruited from the regional trauma system (see the Appendix, which describes the proposed PROMs study design).

Focus groups

The feasibility study was a prospective qualitative quality improvement initiative that utilized a multidisciplinary team of representatives who were invited to participate in a single one-hour virtual focus group. There were multiple focus groups offered to allow for the busy schedules of the various professionals invited. Each focus group was facilitated by the co-investigators with the intent to explore the feasibility of the PROMs study design, which was to be described during the focus groups using a semi-structured interview (Table 2).

**Table 2:** Semi-structured interview guide APP, advanced practice provider; pts, patients

Objective	Main Question	Additional Probes
1. Evaluation of recruitment capability and resulting sample characteristics	Can we recruit appropriate participants?	Feasible and suitable eligibility criteria: Are criteria clear and sufficient or too inclusive or restrictive? Who is the person who determines the eligibility criteria? Would this be an inpatient staff (e.g., resident or APP) or outpatient clinic staff? Obstacles to recruitment: Are orthopedic clinic staff willing to assist with recruitment?
2. Evaluation and refinement of data collection procedures and outcome measures	How appropriate are the data collection procedures and outcome measures for the intended population and purpose of the study?	Who would be the responsible staff to identify eligible/consented patients and provide the survey to the patients? Does the overall survey take a reasonable amount of time [enter number of minutes] or does it burden staff/pts? Do the pts have the capacity to complete the survey? What format would work best in your opinion (e.g., electronic tablets with a REDCap survey link or paper surveys). Electronic tablets: need to be secured, charged, cleaned, distributed, and collected. Paper surveys: need to be distributed, collected, secured, and returned.
3. Evaluation of acceptability and sustainability of intervention and study procedures	Are study procedures and intervention suitable for and acceptable to participants?	Does the survey fit in the workflow of the inpatient and orthopedic clinic? Does the survey distribution and collection take a reasonable amount of time? Would pts be willing to complete the survey during their visit? Is there a better time to implement this study (e.g., season of the year, vaccination time frame, etc.)?
4. Evaluation of resources and ability to manage and implement the study and intervention	Does the research team have the resources and ability to manage the study and intervention?	What research resources are available at each institution (e.g., research support, skills, space, and time)? What equipment is required and can it be utilized appropriately? What is involved in training staff or patients to use the equipment? Are we able to efficiently and effectively manage data entry?

Participant Recruitment and Sampling Strategy

The regional trauma system recruited a multidisciplinary team of professionals (e.g., TPMs, OPMs, orthopedic providers, and orthopedic outpatient clinic staff) to participate in a one-hour virtual focus group by email. One of the project co-investigators sent an email to all of the regional trauma system’s TPMs and asked them to forward the email to their institution’s orthopedic clinic practice managers using a snowball sampling approach to ensure orthopedic staff representation. Within the email, there was a REDCap (Research Electronic Data Capture) link for a survey that gathered information on the interest and availability of staff to participate in the one-hour virtual focus group. Those who were interested in participating were notified of the date and time of the one-hour virtual focus group via email, which included the meeting link.

The study design (see the Appendix) was presented to a multidisciplinary focus group of up to 15 professionals from different trauma centers in the regional trauma system for one one-hour virtual focus group. There were five objectives of the feasibility study with each objective having a main question to answer (Table 1). The focus group was guided using the semi-structured interview guide (Table 2).

Ethical considerations

This study was completed in accordance with the Declaration of Helsinki, and protected the life, health, dignity, right to self-determination, privacy, and confidentiality of personal information of research subjects. After discussion with the Office of Research Administration at our institution (Summa Health), it was determined that this study did not require IRB approval and a non-human subject determination was granted.

Data collection methods, processing, and analysis

This qualitative feasibility study gathered staff feedback using a semi-structured interview guide that facilitated focus groups to ascertain the data. The feasibility study was conducted via a virtual meeting platform in order to comply with social distancing due to the COVID-19 pandemic and encourage participation from multiple institutions. There were a total of three one-hour virtual focus groups, which were audio and visually recorded, which included a total of seven participants. Each audio-recorded focus group was transcribed verbatim. The participant responses were de-identified and pseudonyms were utilized to protect the identity of participants during the analysis of the focus groups. The transcribed focus groups were then uploaded into the NVivo 12 professional software (QSR International, Burlington, MA) for storage and analysis. The analysis followed the first four steps of Moustakas’ modification of the van Kaam method of analysis (Table 3) [16,18,19]. The lead investigator and two co-investigators reviewed all of the coded responses and came to a unanimous consensus regarding the codes before beginning to merge codes which allowed for themes and subthemes of the focus group interviews to emerge. There were a total of three focus groups, which included seven total participants analyzed before thematic saturation was achieved.

**Table 3:** Moustakas' steps of phenomenological analysis

Listing and Preliminary Grouping	Listing Relevant Expressions
Reduction and elimination	Test each expression for two requirements: contains a necessary and sufficient moment of the experience, capable of abstraction and labeling
Clustering and thematizing the invariant constituents	Categorize related experiences into categories and themes
Final identification of the categories and themes	Review the categories and themes against the entire transcript

Data trustworthiness

Triangulation was used as a means of reducing the risk of bias and increasing the trustworthiness of the data. Thematic analysis was achieved using the following steps. First, the two co-investigators read the transcribed focus groups verbatim. Second, each of the co-investigators and lead investigator coded the transcripts together, on separate occasions, and discussed the codes before reaching complete agreement on the appropriate codes. Third, the lead investigator and two co-investigators began collapsing the codes by allowing the categories, subthemes, and finally themes to emerge from the data. The finalized concept map was reviewed by the lead investigator and two co-investigators who unanimously agreed upon the results based on the analysis.

## Results

**Figure 1:**
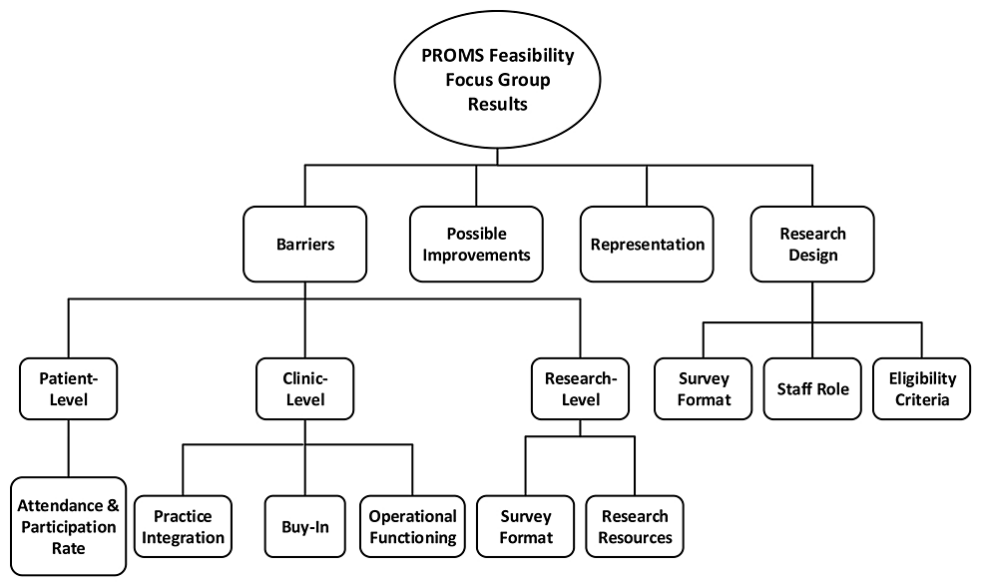
Concept map of themes PROMs, patient-reported outcome measures

There were three focus groups conducted with a total of seven participants. The themes that emerged from qualitative analysis are displayed in Figure 1. The four themes were (1) barriers, (2) possible improvements, (3) representation and (4) research design. Details of each theme, subtheme, and category that prevailed during our analysis can be found in Table 4. Results from each individual theme are presented in greater detail next.

**Table 4:** Data analysis and categorization EHR, electronic health record; ortho, orthopedic; rep, representation

Theme		Subtheme		Category	
Name	Data	Name	Data	Name	Data
Barriers		Patient level	"[Patients] would get mad at the front desk and they would throw the paper at them."	Attendance and participation rate	"I don’t know what the true response would be, but my guess is that it wouldn’t be great."
		Clinic level	"The number of patients who get a follow-up clinic appointment might vary and some places may not have trauma follow-up clinics at all."	Practice integration	"There are a lot of facilities that do not have their own. They have ortho that covers more than one place."
				Buy-in	"Front desk, if they got five people in the line, what are they going to skip?"
				Operational functioning	"[…] We aren’t really in the orthopedic offices to see 20 minutes sounds pretty good I don’t know what their wait times usually are or their work flow."
		Research level		Survey format	"We do all the survey on patient care […] you don’t get a good response because by the time you get to the third one they are like 15 minutes long, it’s horrible."
				Research resources	"At a level I or level II that’s going to be a little bit harder because we have a [researcher]."
Possible improvements	"What if there was an opportunity to build into [EHR … to indicate] patient needs to be provided with a survey."				
Rep	"I want the voice of our trauma center and the orthopedic clinics to be heard for the College."				
Research design	"One thing [regarding implementation], is trauma season tends to be up a little more in the warmer months maybe through October. […] you get a little bit more patients."	Survey format	"Elderly or if they are not comfortable with tablets, [provide] paper resource."		
		Staff role	"Patient to complete through MyChart taking away from a busy practice like mine, front desk to do this."		
		Eligibility criteria	"Even if you could develop some kind of card to give to the orthopedic patient at discharge, that they could present at the orthopedic clinic when they go."		

Barriers

“Barriers” presented as a theme due to participants expressing concern regarding various challenges in executing the PROMs study. Three subthemes emerged from the “barrier” theme: (1) patient level, (2) clinic level, and (3) research level. Within the subtheme of patient-level barriers, interviewed staff expressed concern for challenges faced by patients when completing surveys stating, “It is definitely an obstacle for us [...]. I have been an orthopedic patient here at our facility and fortunately I only had a boot done but there are a lot of people who do not have the abilities to carry.” This could impact the ability of patients to carry a tablet if the surveys were electronic or use a writing instrument if the surveys were in paper form. There were clear concerns for the physical capabilities of patients post-trauma as a barrier for patients. A category that prevailed under patient level was attendance and participation rate. Staff noted a conscious concern for the time patients would spend responding to surveys as an inhibiting factor sharing, “they do surveys all the time on patient care and they’re ridiculous [...]. You don’t get a good response and they are 15 minutes long, it is horrible.”

Additionally, clinic-level barriers regarding implementation were identified. One participant stated, “You know not everyone will have a trauma follow-up clinic [appointment].” A lack of follow-up post-trauma in clinic represents a barrier faced by certain trauma centers. Additionally, three categories emerged under the clinic level that included (1) practice integration, (2) buy-in, and (3) operational functioning. Practice integration was identified as the overall collaboration between the trauma center and the orthopedic practice. Some staff expressed concerns due to the use of a non-hospital affiliated orthopedic practice at their center, and the effort that would be required to connect regarding the eligible patients. In the category of buy-in, a participant noted, “I don’t want to create a bunch of burden on our orthopedic outpatient clinics.” Seeing this proposed study as a burden indicated that there is not a high value placed on data collection, which could impact the commitment from staff. This indicated a concern for clinical staff, from those who were interviewed. The category of operational functioning included aspects related to the functioning of data collection within clinics. One interviewed staff member shared that her practice was busy and implied a potential difficulty for front desk staff to carry out the data collection procedure.

The final subtheme that emerged within “barriers” was at the research level. The categories that prevailed within this subtheme were (1) survey format and (2) research resources. Within the category of survey format, staff expressed different barriers that would be seen in regard to the specific formatting (i.e., electronic vs. paper) of the survey used for data collection. Resources were noted as a potential barrier for some clinics, as many clinics who may not have a dedicated member of the research team embedded in their department may have a harder time carrying out data collection.

Possible improvements

While barriers to this study design were a significant result of the focus groups, “possible improvements” emerged as another theme. Participants expressed ways to improve the operational functioning and integration of data collection for clinic staff. One staff member stated, “Like we said it would be really quick to set them up, ‘here’s the link that takes you to the survey’ or ‘here’s the paper form’ kind of explain to them and then afterwards kind of take that away.” Other participants suggested, “I know we’ve used the QR codes on paper in the past and they were really easy to work with.” Potential improvements focused on patient access to surveys using electronic health record (EHR) patient portals to make implementation smoother and more efficient.

Representation

An additional theme encountered was that of “representation.” One TPM stated, “I want the voice of our trauma center and the orthopedic clinics to be heard for the College.” As discussed in detail above, there are a plethora of barriers that limit the application of the PROMs study in practice. The participants in this study expressed the desire for the ACS to hear their concerns before mandating PROMs, so that trauma program staff are not overwhelmed with an additional data collection responsibility without the required resources to achieve it on top of the required trauma registry data collection.

Research design

Many staff shared concerns about the “research design” of the study, a theme that was further broken down into three subthemes: (1) eligibility criteria, (2) staff role, and (3) survey format. When discussing the implementation of the PROMs study, one staff member shared, “You know, one thing [regarding implementation], is trauma season tends to be up a little more in the warmer months maybe through October. So, I don’t know maybe you get a little bit more patients.” This represents the concern of personnel requirements needed to implement and maintain the study, and how these requirements may change throughout the year as trauma volumes fluctuate.

Within the eligibility criteria subtheme, staff expressed the practical consideration of how they were going to differentiate clinic patients who meet inclusion criteria for the PROMs study from those who are not trauma patients and, therefore, would be excluded from the study. One participant suggested, “Even if you could develop some kind of card to give to the orthopedic patient at discharge, that they could present at the orthopedic clinic when they go.”

Another subtheme that emerged was staff role. This subtheme was particularly multi-dimensional as it is dependent upon each individual practice structure, but still demands some type of consistency to allow for a replicable study. A participant from our study suggested taking responsibility off of the staff completely, stating, “Push it into MyChart [a patient EMR portal] for the patient to complete through MyChart taking away from a busy practice like mine, the front desk to do this.” While we discussed this concept in the possible improvements theme, it clearly related to the staff role subtheme as well.

The final subtheme that emerged under research design was survey format. Again, it became clear that administering a standardized survey across diverse practice styles and patient demographics would be a challenge. A staff member voiced an idea to overcome this stating, “if they are maybe elderly or if they are not comfortable with tablets, maybe providing them just instantly that paper resource and then the staff.” This idea could place additional stress on staff to transfer this information to a standardized electronic format.

## Discussion

The aim of this study was to explore the feasibility of PROMs data collection within a midwestern regional trauma system. The ACS TQIP has shared the importance of PROMs in order to better understand the long-term impact of patient outcomes on recovery [1]. Research clearly supports the long-term impact of behavioral health conditions on trauma patients [11,14]. Furthermore, the existing literature supports the challenges associated with patient survey responses and staff buy-in related to behavioral health assessments [2-5,12,14]. Findings from this feasibility study provide insights into staff perspectives related to PROMs data collection in one trauma region. These focus groups provided an opportunity for staff to share their perceptions of PROMs data collection; the four themes, (1) barriers, 2) possible improvements, (3) representation and (4) research design, and the connection to the existing literature are discussed below.

Practice integration

An interesting and unexpected finding in this study was the impact of orthopedic practice integration into a health system that includes a trauma center. It is known that not all orthopedic practices are employed by healthcare systems that include trauma centers. This raises issues in the execution of PROMs and is another factor that would need to be addressed regarding the communication between trauma services and outpatient orthopedic offices at these practices, if that is the targeted sample population. For the research purposes, this would potentially mean challenges in communicating about patients and getting an orthopedic practice a list of patients who were interested in participating in the PROMs study. For a patient, this could increase confusion if it is unclear that their information will be shared with the orthopedic clinic to follow up with patient surveys. Due to the challenges of coordinating care with an external orthopedic practice, it is easy to see how PROMs may become less of a priority when facing alternative clinical issues.

Possible improvements: QR codes and technology

Under the theme of possible improvements, one participant suggested providing patients with QR codes to administer surveys. This would link patients to a centralized database for PROMs, avoiding additional responsibilities for staff, data storage concerns, and/or responsibility for the maintenance of technology. By providing a QR code, patients could use a personal smart phone or tablet to access the patient surveys, thereby relieving the staff from providing a tablet or paper survey, which they would need to monitor otherwise. Removing the paper survey or tablet would eliminate the need to appropriately account for the storage and security of patient research records or technology. Regardless of the survey format, collecting surveys before the patient arrives has been previously shown to be effective [20]. Using an EHR system or patient EHR portal was also discussed in the focus groups as an option for survey data collection that would not place additional burden on the staff. Survey reminders could be incorporated into EHR or patient EHR portal surveys, which has been shown to increase response rates [2].

Staff buy-in

A key barrier that emerged from the focus groups was the lack of staff buy-in noted among the interviewed professionals. The proposed study was regarded as a potential burden to busy clinics, implicating a lack of value assigned to this issue and an overall lack of support from the community. Despite substantial literature demonstrating the importance of behavioral health screening following traumatic injury, there are a number of system-level barriers to the implementation [6-11]. While the literature on this topic is somewhat mixed, studies appear to have consensus regarding the lack of time and resources available in the medical community to carry out further screening protocols [8,11]. This could be due to the presence of stigma towards behavioral health in the medical community, as well as an overall lack of staff and resources in healthcare settings [13].

In addition to the lack of staff and resources, overseeing PROMs surveys places an increased burden and time constraints on existing administrators without any additional financial incentive. Due to this, the buy-in component of integration has been documented as a potential source of failure previously [20]. In fact, it has been shown that physician and administrative engagement were the strongest predictors of increasing PROMs collection rates, followed by financial incentives [15].

Research design

In regard to the research design of this proposed study, concerns were raised about personnel requirements, difficulties for clinics to carry out screening, and survey format. One particular issue was about the fluctuating levels of trauma intakes throughout the year and the effect this would have on the personnel required to carry out data collection, which has been supported by the literature [21].

Research is inconclusive regarding staff support for electronic data [4]. Researchers found participant response rates were higher for paper survey administration (86%) compared to electronic formats (71%). However, other research has demonstrated no difference between these methods [2]. Paper surveys would place an additional burden on staff, including the requirement for manual data entry, data storage and security, and time for survey completion. However, this may have the potential to increase response rates as they are administered during the visit under staff supervision. EHR linkage, on the other hand, would allow for reminders, central data localization, and automation, but would create problems due to communication issues between healthcare systems. In order to reach a true sample of trauma patients, geriatric patients need to be considered; this includes the challenges some experience with technology leading to the benefit of paper surveys. It is necessary to obtain a standardized survey format that can both produce an optimal patient response rate and avoid placing too much additional stress on staff to carry out survey administration and collection. This is difficult due to the varying levels of resources available to different clinics.

Limitations

The limitations of this study include those inherent to qualitative methodologies and others that were specific to this study. The first limitation was the previously established relationship the lead investigator had with some of the focus group participants. This was unavoidable due to the limited skill set of qualitative researchers to facilitate focus groups within the region’s trauma centers. In order to limit the potential bias, the two co-investigators transcribed the focus groups and the team coded the interviews separately. The second limitation would be the small sample size of respondents, which consisted of TPMs and OPMs. Even for a qualitative study, the sample size was small and indicated a limited amount of feedback from even the regional trauma system. Additionally, due to the focus on orthopedic trauma patients, some of the information obtained from the feasibility study may not be applicable to non-orthopedic trauma patients. A third limitation was the pre-existing relationship focus group participants had with the co-investigators, which may have impacted the responses. Last, as with all qualitative studies, this study does not produce generalizable results, but rather insights into the feedback of one regional trauma system related to a PROMs study for orthopedic trauma patients.

## Conclusions

This qualitative feasibility study demonstrated the strengths and weaknesses of a proposed PROMs study for trauma centers to expand to longitudinal data collection. The desire for the ACS to expand data collection through PROMs is not only reasonable, but also imperative to continuously improve trauma care for injured patients. The implementation of an expanded data collection plan may enhance the ability to improve the short- and long-term health outcomes in patients, but the feasibility study emphasized several barriers for trauma centers and the ACS to consider prior to encouraging or mandating long-term data collection. Awareness regarding the barriers may provide individual trauma centers, regional trauma systems, and TQIP insights into ways to strategically develop opportunities to implement successful long-term data collection for patients.

## Appendices

Proposed PROMs study design

The patient-reported outcome measures (PROMs) study is the project that the feasibility study will be answering questions on to determine its practicality and the barriers that a region’s trauma centers may face if it was to be initiated. The PROMs study is a prospective, descriptive multi-institutional study that uses surveys to ascertain information regarding orthopedic trauma patients’ physical and behavioral health during their recovery.

Patient inclusion criteria will be as follows: (1) would meet the eligibility of the trauma registry inclusion criteria, (2) have an orthopedic injury (i.e., operative fractures), and (3) have an appointment scheduled to follow up with an orthopedic provider in an outpatient orthopedic clinic. For adult trauma centers, this will be patients aged 18 years and older; for pediatric trauma centers, this will be patients who are at least 11 years old, and parental consent.

Patient exclusion criteria will be as follows: (1) were not treated by an orthopedic provider, (2) did not experience an orthopedic injury, (3) do not have an orthopedic outpatient clinic appointment scheduled, (4) code status was 'do not resuscitate - comfort care only' (DNR-CCO), and (5) limited cognitive functioning as defined by having a traumatic brain injury, dementia, or other cognitive impairment as determined by a medical professional.

The patient will be identified upon discharge from their initial hospital admission for their traumatic injury, which will be communicated to the outpatient orthopedic clinic in some manner, which is yet to be determined. During the outpatient orthopedic follow-up clinic appointment, the front desk will ask eligible patients to complete a REDCap survey using an electronic tablet prior to their orthopedic provider coming in for the appointment. The survey will take approximately 15 minutes to complete. Patients will be asked to participate in survey completion at their three-month post-injury orthopedic follow-up appointment.
